# Comparative Models of Biological and Social Pathways to Predict Child Growth through Age 2 Years from Birth Cohorts in Brazil, India, the Philippines, and South Africa

**DOI:** 10.1093/jn/nxy101

**Published:** 2018-07-13

**Authors:** Linda M Richter, F Mark Orkin, Gabriela D Roman, Darren L Dahly, Bernardo L Horta, Santosh K Bhargava, Shane A Norris, Aryeh D Stein, COHORTS investigators

**Affiliations:** 1 DST-NRF Centre of Excellence in Human Development; 2 MRC/Wits Developmental Pathways for Health Research Unit, Faculty of Health Sciences, University of the Witwatersrand, Johannesburg, South Africa; 3 Institute of Criminology, University of Cambridge, Cambridge, United Kingdom; 4 HRB Clinical Research Facility—Cork, University College Cork, Cork, Ireland; 5 The Centre of Epidemiological Research, Universidade Federal de Pelotas, Pelotas, Brazil; 6 Sunder Lal Jain Hospital, New Delhi, India; 7 Hubert Department of Global Health, Rollins School of Public Health, Emory University, Atlanta, GA

**Keywords:** infant, growth failure, birth cohort, structural equation modeling, longitudinal model, social, environmental, biological

## Abstract

**Background:**

Early growth faltering accounts for one-third of child deaths, and adversely impacts the health and human capital of surviving children. Social as well as biological factors contribute to growth faltering, but their relative strength and interrelations in different contexts have not been fully described.

**Objective:**

The aim of this study was to use structural equation modelling to explore social and biological multidetermination of child height at age 2 y in longitudinal data from 4 birth cohort studies in low- and middle-income countries.

**Methods:**

We analyzed data from 13,824 participants in birth cohort studies in Brazil, India, the Philippines, and South Africa. We used exploratory structural equation models, with height-for-age at 24 mo as the outcome to derive factors, and path analysis to estimate relations among a wide set of social and biological variables common to the 4 sites.

**Results:**

The prevalence of stunting at 24 mo ranged from 14.0% in Brazil to 67.7% in the Philippines. Maternal height and birthweight were strongly predictive of height-for-age at 24 mo in all 4 sites (all *P* values <0.001). Three social-environmental factors, which we characterized as “child circumstances,” “family socioeconomic status,” and “community facilities,” were identified in all sites. Each social-environmental factor was also strongly predictive of height-for-age at 24 mo (all *P* values <0.001), with some relations partly mediated through birthweight. The biological pathways accounted for 59% of the total explained variance and the social-environmental pathways accounted for 41%. The resulting path coefficients were broadly similar across the 4 sites.

**Conclusions:**

Early child growth faltering is determined by both biological and social factors. Maternal height, itself a marker of intergenerational deprivation, strongly influences child height at 2 y, including indirect effects through birthweight and social factors. However, concurrent social factors, many of which are modifiable, directly and indirectly contribute to child growth. This study highlights opportunities for interventions that address both biological and social determinants over the long and short term.

## Introduction

An estimated 30% of children in low- and middle-income countries (LMICs) are stunted (1); malnutrition is the underlying cause of death of approximately one-third of children under age 5 y (2) and, amongst surviving children, growth faltering is associated with adverse medium- and long-term health and human capital consequences, including delayed early development, lower cognitive performance and school

achievement (3), higher rates of child conduct disorders and hyperactivity (4), an increased risk of failing a grade and a reduction in overall years of schooling (5), impaired physical growth and shorter stature in adulthood (6), decreased earnings and assets in adulthood (7), and increased risk of obesity (8) and cardiometabolic disease (9). Shorter women have babies with lower birthweight and a greater risk of being stunted and dying than do children of women of average height (10). These disadvantages may be transmitted to the next generation through suboptimal growth (11) and human development (12), entrenching cycles of disadvantage.

Increases in height in high-income countries over long periods of time are ascribed largely to economic development (13). Correspondingly, the underlying causes of growth failure are poverty, inequality, food insecurity, and lack of access to essential services (2). Both ecologic (14) and epidemiologic (15, 16) frameworks propose that these underlying causes operate through intermediate and immediate factors, shaping the proximal environment and experiences of children, causing inadequate nutrition and diarrhea which result in growth failure. To date, these frameworks, and the direction of their effects, have been examined mainly through cross-sectional data, from which it is not possible to determine temporal sequences or make causal inferences (17). Prior analyses of determinants and consequences of growth failure from longitudinal data have considered social and environmental variables such as socioeconomic status (SES), maternal age and education, birth order, marital status, and urban/rural residence primarily as confounders rather than co-determinants of early child growth (11, 18, 19), and its consequences on adult health and wellbeing (3, 20). Yet countless studies have shown that a range of social and environmental factors are independently associated with health outcomes, including infant growth (21). Despite this, multidetermination of growth faltering has not been systematically studied, nor how it might vary by context.

In this paper we use unique longitudinal data from birth cohorts in 4 LMICs to examine the relative contributions of a wide set of both biological and social variables as co-determinants of infant growth. We use structural equation modeling to describe the direct and indirect paths through which these biological and social factors predict child height at age 2 y.

## Methods

COHORTS (the Consortium of Health Orientated Research in Transitioning Societies) is a collaboration among 5 large and long-running birth cohort studies in LMICs (22). The 5 birth cohorts are: the 1982 Pelotas (Brazil) Birth Cohort (23); the Institute of Nutrition of Central America and Panama Nutrition Trial Cohort (INTC; Guatemala) (24); the New Delhi (India) Study (25); the Cebu Longitudinal Health and Nutrition Survey (CLHNS; Cebu, Philippines) (26); and the Birth to Twenty Plus (Bt20+; South Africa) cohort (27).

Data from 4 of the 5 cohorts, Brazil, India, the Philippines, and South Africa, were included in this analysis. The data from Guatemala were excluded because this study was a randomized community trial, designed to minimize social variation relevant to the outcome of a nutrition intervention on children's growth. The processes for identifying Guatemalan villages hence controlled for the social variables we were examining. The cohorts vary by country, epoch, and SES, providing different contexts for the examination of determinants of child growth. The Brazilian study enrolled 5914 children from all socioeconomic groups born in Pelotas’ maternity hospital in 1982, which covered >99% of all births in the city. The Indian cohort enrolled 8181 babies born to married, mostly middle-class, women in a defined area of New Delhi between 1969 and 1972. The Philippine cohort enrolled pregnant women from all socioeconomic groups living in 33 randomly selected, mostly urban (75%) neighborhoods in Cebu between 1983 and 1984 (3080 infants). The South African cohort enrolled mostly poor black pregnant women living in a defined urban area of Johannesburg in 1990 (3273 infants).

All the studies were reviewed and approved by an appropriate ethics committee or institutional review board.

### 

#### Measures

Birthweight was measured in grams in hospitals and clinics at delivery in Brazil and South Africa, in hospitals or at home by birth attendants in the Philippines, and in the community within 72 h of birth in India. Maternal height was measured by a stadiometer and recorded to the nearest 0.1 cm following standard procedures at cohort enrolment in Brazil and the Philippines, and at birth or in early childhood in India and South Africa. Height-for-age was measured at around 24 mo of age, with some variability among sites. In all cohorts, measurements were converted to height-for-age *z* scores with reference to WHO standards and with the use of children's exact age at measurement (28).

The specific social factors used in this analysis were selected based on their commonality across the 4 birth cohort sites and their prior identification as determinants of height in childhood (29). They include maternal and paternal schooling, maternal age at the birth of the child, marital status, wealth (an index calculated from a list of pertinent assets), annual income (per capita), social class (paternal occupation), household crowding (ratio of people per room), sex, birth order, child dependency (ratio of children aged <18 y to adults), and health utilization, sanitation, and access to safe water. The variables were defined in the same way across cohorts except that wealth, social class, and health service utilization, sanitation, and access to water were coded into site-specific ordinal scales of 3 or 5 categories. Birth order, the dependency ratio, and the crowding ratio were reverse-coded to represent more optimal conditions as higher scores.

#### Analysis

We compiled a common dataset from data provided by the 4 birth cohort studies. Participants from the 4 sites were included in the analysis if they had child height at 24 mo. Differences between means from the pooled data between cases included and excluded were small according to Cohen's *d*, except for paternal schooling, social class, annual income, and birth weight, which were small to middling (**Supplemental Table 1**).

Child birthweight (10) and maternal height (11) were investigated as biological determinants of height-for-age at 24 mo. The social variables pertain to the first 2 y of a child's life.

**FIGURE 1:**
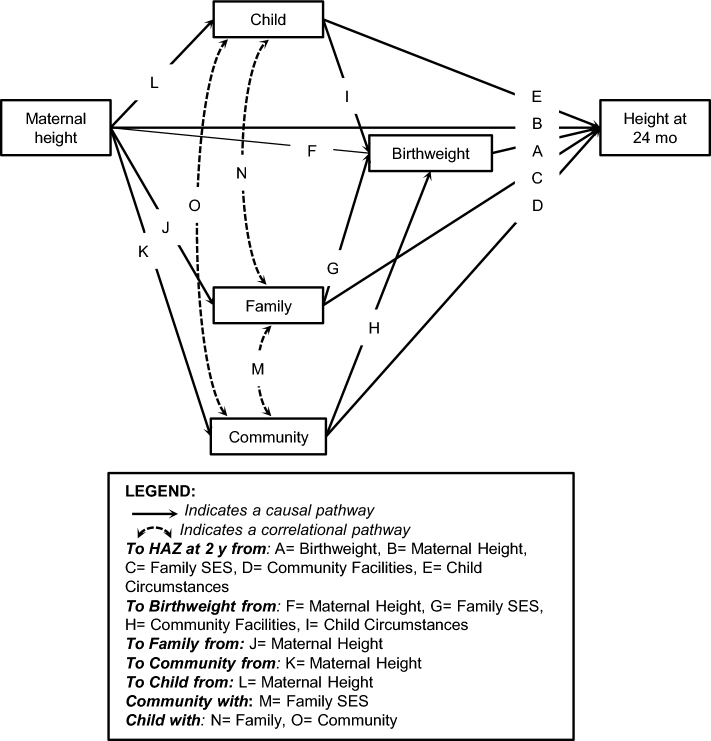
Model depicting direct and indirect paths from social and biological determinants to child height. Letters correspond to paths in Table 3. HAZ, height-for-age *z* score; SES, socioeconomic circumstances.

Exploratory structural equation modeling (ESEM) (30) was used to review the loading of the social variables onto 2, 3, and 4 factors in relation to specified paths to the outcome. ESEM combines exploratory and confirmatory factor analytic strategies, which is helpful when prior theory is limited. Modelling decisions were based on a biologically driven conceptual framework (**Figure 1**) in which maternal height was considered to be exogenous to the child's birth circumstances (including family socioeconomic circumstances and community facilities) since maternal height primarily reflects the biological and social conditions when the mother herself was born and raised as a young child (3).

The resulting measurement models were tested for invariance across the 4 sites by means of multigroup confirmatory factor analysis, with the use of a composite sample weighted equally by site. The factor scores from the measurement models were included with the biological determinants (maternal height and birthweight), together with the outcome (height-for-age *z* score at 24 mo) in a hypothesized path model (Figure 1) that tested the main research question about the relation among predictors and their relative strength in transmission of growth failure (11). From the final model, we computed the total direct and indirect effects from predictors to outcome.

Models were validated by a split-sample approach. Initial modelling was done with the use of a sample of two-thirds of the data, randomly sampled from each site, and the final model was checked against the remaining hold-out sample. As shown in **SupplementalTables 1and2**, when the measurement and path models were run with the one-third “hold-out” sample, model fit criteria were all good [root mean square error of approximation (RMSEA) ≤ 0.05, Comparative Fit Index (CFI) ≥ 0.95, and Tucker-Lewis Index (TLI) ≥ 0.95], except for RMSEA for the path model of Brazil, which was fair (0.07) (31). Even the smallest sample, that from South Africa, was >20 times larger than the number of parameters being estimated, reducing the chances of substantial model overfit to data (31). Missing data were accommodated by full information maximum likelihood, which returns unbiased parameter estimates when data are missing at random, conditional on the variables included in the model (32, 33). Fit statistics are reported for RMSEA, CFI, and TLI. All analyses were conducted with SPSS (version 21, IBM Corporation) and Mplus (version 7.1, Muthén & Muthén).

## Results

### 

#### Description of the sample

Of the 13,824 participants in the analytic sample, 4836 were from Brazil, 5342 from India, 2504 from the Philippines, and 1142 from South Africa. The proportion of missing values was <10% for all variables in Brazil and the Philippines except maternal age at index birth (33% and 11%, respectively); <25% for 10 variables and 25–50% for 3 in South Africa; and <25% for 2 variables, 25–50% for 8 variables, and 51–75% for 3 variables in India. However, covariance coverage was always greater than the default Mplus minimum of 10% of data present for each pair of variables (30).

The cohorts differ by time period, country wealth classification, and enrollment by social class: India, a low-income country at the time in the early 1970s, enrolled mainly middle-class families; Brazil, a middle-income country, and the Philippines, a low-income country at enrollment in the mid-1980s, enrolled all classes; South Africa, a middle-income country in 1990, enrolled mainly poor African families. Except for the Philippines, participants were sampled from predominantly urban areas.

Anthropometric and social characteristics are shown in **Table 1**. South African mothers were considerably taller than those in India and the Philippines. Prevalence of low birthweight was highest in India; stunting at 24 mo was highest in the Philippines and lowest in Brazil. Median birth order was highest in India and maternal schooling was highest in South Africa. Marriage was lowest in South Africa. Households in both Brazil and South Africa had relatively good access to sanitation and safe water, whereas only 5.9% of the Philippine sample had access to flush toilets.

**TABLE 1:** Selected characteristics of the study sample, by site (total *n* = 13,824)^1^

	Brazil (*n* = 4836)	India (*n* = 5342)	Philippines (*n* = 2504)	South Africa (*n* = 1142)
Maternal height, cm	156.4 ± 6.1	152.1 ± 5.5	150.6 ± 5.0	158.7 ± 6.5
Maternal age at birth of child, y	25.8 ± 6.1	25.9 ± 5.2	26.3 ± 6.0	26.0 ± 6.1
Maternal schooling, y	6.48 ± 4.19	5.23 ± 4.62	7.11 ± 3.31	9.55 ± 3.00
Paternal schooling, y	6.88 ± 4.27	10.7 ± 4.97	7.33 ± 3.50	10.59 ± 2.83
Marital status at birth of child				
Married	91.8	99.8	97.5	43.5
Birth order				
1	39.3	17.7	22.3	36.6
2	28.1	25.5	22.5	30.2
3	16.3	22.2	19.4	17.7
≥4	16.3	34.6	35.8	15.5
Child dependency ratio (children aged <18 y per adult)	1.21 ± 0.87	1.28 ± 0.76	1.49 ± 0.94	0.86 ± 0.62
Crowding ratio (people per room)	2.94 ± 1.40	4.41 ± 1.97	3.15 ± 1.84	3.02 ± 1.63
Social class^2^				
1 (Lowest)	43.1	1.8	11.5	22.0
2	30.4	10.6	21.7	35.9
3	5.2	21.8	50.5	11.0
4	6.4	49.7	7.7	12.9
5 (Highest)	14.8	15.9	3.8	2.1
Income^2^				
1 (Lowest)	22.1	20.3	20.0	23.3
2	18.5	22.1	20.0	29.6
3	19.6	17.5	20.0	13.6
4	22.1	20.9	20.0	19.7
5 (Highest)	17.8	19.3	20.0	13.8
Use of health services				
Low	32.6	29.1	42.3	17.9
Medium	34.7	30.0	51.3	52.0
High	32.8	40.9	6.5	30.2
Toilet type				
None	0.6	22.7	33.2	0.0
Some	20.2	39.8	60.9	21.8
Flush	79.1	37.5	5.9	78.2
Access to safe water				
Worst	4.7	17.7	14.2	0.0
Intermediate	18.6	47.8	72.4	45.1
Best	76.8	34.4	13.4	54.9
Sex				
Female	48.6	47.9	47.0	51.4
Birthweight, kg	3.19 ± 0.57	2.79 ± 0.44	2.99 ± 0.44	3.07 ± 0.51
Height at 24 mo, cm	80.7 ± 4.9	80.5 ± 3.9	79.2 ± 3.7	83.1 ± 4.1
Stunted at 24 mo	14.0	46.2	67.7	26.2

^1^Data are means ± SDs or percentages.

^2^Social class and income categories are site-specific, based on a 5-point scale.

**TABLE 2:** Standardized factor loadings of social and environmental factors from measurement models across 4 sites, and model fit statistics^1^

					Model fit statistics^2^		
	Brazil	India	Philippines	South Africa	RMSEA	CFI	TLI
Factor 1: Family socioeconomic status					0.03	1.00	1.00
Maternal schooling	0.90 ± 0.02	0.82 ± 0.02	0.89 ± 0.02	0.75 ± 0.02			
Paternal schooling	0.74 ± 0.01	0.66 ± 0.02	0.70 ± 0.02	0.68 ± 0.02			
Income quintile	0.75 ± 0.01	0.74 ± 0.02	0.57 ± 0.02	0.51 ± 0.04			
Social class	0.85 ± 0.01	0.51 ± 0.04	0.36 ± 0.02	0.37 ± 0.04			
Factor 2: Community facilities					0.08	1.00	0.99
Toilet type	0.81 ± 0.02	0.90 ± 0.03	0.87 ± 0.04	0.90 ± 0.04			
Access to safe water	0.98 ± 0.02	0.77 ± 0.03	0.74 ± 0.03	0.93 ± 0.04			
Use of health services	0.13 ± 0.03	0.37 ± 0.03	0.50 ± 0.03	0.03 ± 0.06			
Factor 3: Child circumstances					0.02	1.00	0.99
Child dependency ratio	0.68 ± 0.02	0.70 ± 0.02	0.71 ± 0.04	0.45 ± 0.03			
Crowding ratio	0.34 ± 0.01	0.44 ± 0.01	0.26 ± 0.01	0.28 ± 0.02			
Birth order	0.80 ± 0.02	0.94 ± 0.02	0.79 ± 0.03	0.73 ± 0.04			
Maternal age at birth of index child	0.24 ± 0.02	0.37 ± 0.02	0.29 ± 0.02	0.24 ± 0.02			

^1^Values are SFLs ± SEs. The slight variation of a given standardized loading across sites arises from the variance differences among the sites. All loadings are *P *< 0.001. CFI, Comparative Fit Index; RMSEA, root mean square error of approximation; SFL, standardized factor loading; TLI, Tucker-Lewis Index.

^2^Model fit criteria were good (RMSEA ≤ 0.05, CFI ≥ 0.95, and TLI ≥ 0.95), except RMSEA ≤ 0.075 for Factor 2 which was fair.

We combined data from males and females because the differences in the outcome were small and combining the data simplified the analysis. We dropped from the analysis marital status from Brazil, India, and the Philippines because >90% were married, and wealth in South Africa because lack of variation prevented model convergence.

#### Factors

Three social factors emerged from the ESEM analysis, which we labelled *Child Circumstances*, *Family SES*, and *Community Facilities.* The standardized loadings from cross-site measurement modelling are set out in **Table 2**, with SEs and *P* values, and show appreciable factorial invariance (31) across sites. All 3 model fit statistics were good in all instances, i.e., RMSEA ≤ 0.05, CFI ≥ 0.95 and TLI ≥ 0.95, with the single exception that RMSEA = 0.08 was fair for Factor 2.

For *Child Circumstances*, the birth order and child dependency ratios loaded strongly in all sites; the loading of child dependency was lowest in South Africa. For *Family SES*, maternal and paternal schooling loaded highly in all 4 sites, with income quintile loading moderately; social class varied the most. For *Community Facilities*, toilet and water loaded strongly in all 4 sites, but access to health facilities loaded weakly.

#### Path models

The path models were closely similar across sites (**Table 3**; **Supplemental Figure1**A–D) and all showed good model fit for all 3 statistics, with the single exception that TLI = 0.94 was fair for South Africa. The model also fitted well in a pooled model (Supplemental Figure 1E) with the use of an equally weighted sample of all 4 sites, notwithstanding the greater variances involved. An alternative model was tested in the 4 sites (34), with maternal height and the 3 social constructs all treated as exogenous and correlated. This also showed good fit in all 4 sites. Nonsignificant paths were set to zero in the final models because there was little variance in the variables that led to these paths.

**TABLE 3:** Standardized path coefficients and path model fit statistics, by site and pooled across sites^1^

Path label in Figure 1^2^	Brazil	India	Philippines	South Africa	Pooled weighted
To HAZ at 2 y from					
A: Birthweight	0.33*** ± 0.01	0.29*** ± 0.03	0.23*** ± 0.01	0.28*** ± 0.04	0.27*** ± 0.01
B: Maternal height	0.24*** ± 0.02	0.24*** ± 0.02	0.22*** ± 0.02	0.24*** ± 0.04	0.32*** ± 0.01
C: Family SES	0.17*** ± 0.02	0.32*** ± 0.03	0.17*** ± 0.03	—^3^	0.08*** ± 0.01
D: Community facilities	0.14*** ± 0.02	0.10*** ± 0.03	0.11*** ± 0.03	—	0.25*** ± 0.01
E: Child circumstances	0.11*** ± 0.02	0.08* ± 0.08	0.20*** ± 0.02	0.11* ± 0.04	0.18*** ± 0.01
To birthweight from					
F: Maternal height	0.18*** ± 0.02	0.15*** ± 0.03	0.17*** ± 0.02	—	0.21*** ± 0.01
G: Family SES	—	0.09* ± 0.04	0.08** ± 0.03	0.15*** ± 0.05	—
H: Community facilities	0.08*** ± 0.02	0.11* ± 0.04	—	—	0.17 ± 0.02
I: Child circumstances	–0.07*** ± 0.02	–0.21*** ± 0.04	–0.18*** ± 0.03	–0.18*** ± 0.05	–0.11*** ± 0.02
To family from					
J: Maternal height	0.25*** ± 0.02	0.20*** ± 0.03	0.17*** ± 0.02	0.12* ± 0.05	0.25*** ± 0.01
To community from					
K: Maternal height	0.15*** ± 0.02	0.07* ± 0.03	0.09*** ± 0.02	—	0.18*** ± 0.02
To child from					
L: Maternal height	0.08*** ± 0.02	—	—	—	0.21*** ± 0.01
Community with					
M: Family SES	0.44*** ± 0.01	0.34*** ± 0.03	0.58*** ± 0.02	0.19*** ± 0.05	0.05*** ± 0.01
Child with					
N: Family	0.25*** ± 0.02	0.47*** ± 0.03	0.31*** ± 0.02	0.24*** ± 0.05	0.27*** ± 0.01
O: Community	0.19*** ± 0.02	0.18*** ± 0.03	0.20*** ± 0.02	—	0.19*** ± 0.01
Model fit statistics^4^					
RMSEA	0.03	0.02	0.03	0.04	0.02
CFI	1.00	1.00	0.998	0.97	1.00
TLI	0.98	0.99	0.99	0.94	0.99
R^2^	0.33 ± 0.01	0.37 ± 0.02	0.26 ± 0.02	0.14 ± 0.03	0.44 ± 0.01

^1^Values are path coefficients ± SEs. ***P < 0.001, **P < 0.01, *P < 0.05. CFI, Comparative Fit Index; HAZ, height-for-age *z* score; RMSEA, root mean square error of approximation; SES, socioeconomic status; SFL, standardized factor loading; TLI, Tucker-Lewis Index.

^2^Letters correspond to paths in Figure 1.

^3^Denotes nonsignificant pathway (*P *> 0.05) set to 0.

^4^Fit statistics computed with nonsignificant paths set to 0.

In all sites, maternal height had a direct path to child height at age 2 y, as well as a mediated path through birthweight (except in South Africa). Maternal height also had mediated paths through the social variables directly onwards to child height at age 2 y: through all 3 social variables in Brazil and in the site-pooled model, through *Family SES* and *Community Facilities* in India and the Philippines, but through none of the social variables in South Africa. Additionally, there were several indirect paths from the social variables through birthweight to child height at age 2 y: e.g., from *Child Circumstances* in all 4 sites. All path coefficients in all 4 models were positive except that between *Child Circumstances* and birthweight.

**Table 4** shows the standardized total effects of the biological and social determinants on infant growth. The total effects of the biological variables were slightly stronger than the total effects of the social factors, except for South Africa where the total effect of the social factors was notably weak.

**TABLE 4:** Standardized direct and total indirect effects of biological and social-environmental pathways on child linear growth at age 2 y, in 4 sites and pooled across sites^1^

	Brazil		India		Philippines		South Africa		Pooled weighted	
	Direct	Indirect	Direct	Indirect	Direct	Indirect	Direct	Indirect	Direct	Indirect
Biological pathways										
Birthweight	0.33*** ± 0.01	0.00	0.29*** ± 0.03	0.00	0.23*** ± 0.02	0.00	0.28*** ± 0.04	0.00	0.27*** ± 0.01	0.00
Maternal height	0.24*** ± 0.02	0.13*** ± 0.01	0.24*** ± 0.02	0.12*** ± 0.02	0.22*** ± 0.02	0.08*** ± 0.01	0.24*** ± 0.04	0.01 ± <0.01	0.32*** ± 0.01	0.16*** ± 0.01
Subtotal^2^	0.69		0.65		0.53		0.52		0.76 (59)	
Social-environmental pathways										
Family SES	0.17*** ± 0.02	0.00	0.32*** ± 0.03	0.03* ± 0.01	0.17*** ± 0.03	0.02** ± 0.01	0.00	0.04* ± 0.02	0.08*** ± 0.01	0.00
Community facilities	0.14*** ± 0.02	0.02*** ± 0.01	0.10*** ± 0.03	0.03** ± 0.01	0.11*** ± 0.03	0.00	0.00	0.00	0.25*** ± 0.01	0.05*** ± 0.01
Child circumstances	0.11*** ± 0.02	–0.02*** ± 0.01	0.08* ± 0.03	0.06*** ± 0.01	0.20*** ± 0.02	–0.04*** ± 0.01	0.11* ± 0.04	–0.05** ± 0.02	0.18*** ± 0.01	–0.03*** ± <0.01
Subtotal^2^	0.42		0.49		0.46		0.11		0.53 (41)	
Total^2^	1.11		1.14		0.99		0.63		1.29 (100)	

^1^Values are direct and total indirect effects ± SEs unless otherwise indicated. SEs smaller than 0.01 are denoted as <0.01; ***P <* *0.001, **P <* *0.01, *P <* *0.05. SES, socioeconomic status.

^2^The subtotal percentages in the pooled sample are 0.76 (59%) or 0.53 (41%) of the total 1.29 (100%) for the biological and social-environmental pathways, respectively.

## Discussion

In this paper, we used unique longitudinal data from 4 of the 5 COHORTS studies, with their varying social and temporal contexts, to examine both biological and social variables as co-determinants of early child growth and growth faltering. We used ESEM, confirmatory factor analysis, and path analysis to examine direct and indirect paths from immediate and intermediate determinants to child linear growth, without assuming which social variables relate to child growth, how they fit into constructs, or which patterns of relations exist among identified determinants.

The results confirm the consistency and strength of the biological pathways to child height at 2 y, directly from maternal height and indirectly through birthweight. This constitutes a core set of determinants of child linear growth at age 2 y. However, strong consistency also emerged in the way the social variables cohered and the similarity and stability of their factor coefficients, despite temporal and economic differences across the sites. Maternal and paternal schooling are strong in *Family SES* in all sites; toilet and water quality in *Community Facilities*; and birth order and child dependency ratio in *Child Circumstances*. Methodologically, the consistency across sites corroborated the causal mechanism, broadly understood, as intimated by the pathways (35). Substantively, these findings support ecologic (14) and epidemiologic (16) models of health and wellbeing, which propose that distal factors such as maternal height exert their influence through proximal factors at the family and community levels that structure day-to-day experiences of children which influence growth.

Also broadly similar across sites are the mediated paths from maternal height operating through all or some of the social mediators. The impacts of the social variables are themselves both direct and mediated through birthweight. These pathways indicate that the impact on child growth of intergenerational advantage or disadvantage, as manifested in maternal height, operates not only biologically (directly and mediated via birthweight), but also through the social context in which women live. Considering the total effects of these direct and indirect paths, the effect of the social variables on child height at 2 y of age is almost as large as the biological variables, except for South Africa.

The consequence of these interrelations is that social and biological predictors are both distal and proximal determinants. Thus, in the best-fit models, both social and biological determinants have direct and indirect effects on child linear growth. These interrelations were robust across 4 varying contexts. Child growth faltering is a consequence of combinations of the social and biological, inextricably bound together by the mother's biological and social history and the current circumstances of the child and family; indeed, the latter are themselves the outcome of the mother's history as much as contemporary influences. Suitable analytic tools enable us to transcend binary thinking about social and biological science and adopt systems approaches to epidemiology and intervention (36). Such thinking makes it clear that interventions, whether social or biological, must take account of both multidetermination and context (17).

Caveats to the findings and conclusions are those common to all observational data. The analysis was limited to data that were commonly collected in the 4 sites between 25 and 45 y ago. We excluded the Guatemalan sample from the analysis because the processes for identifying villages to be included in the trial controlled for the social variables we were interested in examining. Nonetheless, the variables included in the analysis have all been shown in prior studies to be salient to the determination of early child growth (37). Some variability across sites may be due to lack of measurement resolution and lack of data. Missing data were dealt with analytically.

Two particular variations warrant comment. First, the negative path between better *Child Circumstances* and birthweight seemingly contradicts the positive path between better *Child Circumstances* and child height at 2 y. This finding can be accounted for by the fact that first-born infants are typically lighter than later-born children, but taller at age 2 y (18), and are necessarily born into households with lower dependency and crowding ratios.

Second, although the findings are strong in showing appreciable similarity across countries in both direct and indirect social and biological pathways, each site had ≥1 path that was not significant. In South Africa multiple paths were not significant; in fact, there was no indirect path between maternal and child height through social variables. The South African cohort was recruited in Soweto, a dense urban area adjacent to Johannesburg where residents have near-universal access to secondary schooling and relatively good provision of water and sanitation. Although this is not representative of many rural areas of South Africa, the finding suggests that when social conditions such as maternal schooling, water, and sanitation reach a particular level, social variables no longer exert a strong differential effect on child growth. In the Philippines, by contrast, where schooling and access to community facilities were much lower and more variable, social determinants of child height at age 2 y had equal weight to the biological determinants.

The analysis resonates with the call for systems thinking in public health and the design of interventions that respond to both multidetermination and social context. Using unique longitudinal data and subjecting them to modelling, we highlight the multidetermination of child linear growth through age 2 y. We confirm the strong determining role of maternal height and birthweight in child growth faltering, and we identify the substantial concurrent influence of social factors, both directly and in mediating the biological effects. Moreover, we demonstrate invariant configurations of social factors across different contexts of time and location consistent with an ecologic model of causation of growth outcomes. Both social and biological factors, operating in the present and in the past, are levers for child growth, requiring cyclic intergenerational investments and interventions to reduce stunting in early childhood.

## Supplementary Material

Supplement File

## Notes

The analysis in this paper was supported by a Bill and Melinda Gates Foundation grant to COHORTS (OPP1164115). Funding for the individual cohorts was as follows: Pelotas Birth Cohort (Brazil): Wellcome Trust; New Delhi Birth Cohort Study (India): Indian Council of Medical Research, US National Center for Health Statistics, Medical Research Council (United Kingdom), and British Heart Foundation; Cebu Longitudinal Health and Nutrition Study (the Philippines): US NIH; Birth to Twenty (South Africa): Wellcome Trust, Human Sciences Research Council, South African Medical Research Council, University of the Witwatersrand, and the DST-NRF Centre of Excellence in Human Development.

Author disclosures: LMR, FMO, GDR, DLD, BLH, SKB, SAN, and ADS, no conflicts of interest.

Supplemental Tables 1 and 2 and Supplemental Figure 1 are available from the “Supplementary data” link in the online posting of this article and from the same link in the online table of contents at https://academic.oup.com/jn/.

Abbreviations used:

CFI: Comparative Fit Index
COHORTS: Consortium of Health Orientated Research in Transitioning Societies
ESEM: exploratory structural equation modelling
LMIC: low- and middle-income country
RMSEA: root mean square error of approximation
SES: socioeconomic status
TLI: Tucker-Lewis Index
