# Supplementary material for: Comparative Models of Biological and Social Pathways to Predict Child Growth through Age 2 Years from Birth Cohorts in Brazil, India, the Philippines, and South Africa
Source: J Nutr. 2018 Jul 13;148(8):1364–71. doi: 10.1093/jn/nxy101 (PMC6075183; doi:10.1093/jn/nxy101)
Supplement: Supplement File [file nxy101_supplement_file.pdf]

SUPPLEMENTAL TABLE 1. Differences in means between cases included and excluded

	HAZ present Mean[2]	HAZ missing Mean[2]	Cohen's d[1]
Maternal schooling	6.6	5.9	0.17
Paternal schooling	6.9	5.4	0.40
Income quintile	3.0	2.4	0.40
Social class	2.2	1.7	0.36
Toilet type	1.4	1.4	0.03
Access to safe water	1.4	1.4	0.08
Use of health services	1.0	1.0	0.09
Child dependency ratio	1.3	1.3	0.01
Crowding ratio	0.4	0.4	0.10
Birth order	0.6	0.6	0.01
Maternal age at birth of index child	26.0	24.8	0.20
wazwho0[3]	-0.2	-0.7	0.36
Matht	156.5	156.0	0.09

[1] 0.2 is "small", 0.5 is "medium", 0.8 is "large"
[2] Units defined in Methods
[3] Wazwho0 = weight-for-age z-score WHO standards
[4] Matht = Maternal height

SUPPLEMENTAL TABLE 2. Model fit statistics of measurement and path models across four sites, using one-third "hold-out" sample

Model fit statistics	RMSEA	CFI	TLI	R-squared (SE)
Measurement models[1]				
Factor 1	0.03	1.00	1.00	
Factor 2	0.00	1.00	1.00	
Factor 3	0.04	0.99	0.97	
Path models[1]				
Brazil	0.07	1.00	0.93	0.38 (0.02)
India	0.00	1.00	1.00	0.36 (0.03)
Philippines	0.01	1.00	0.99	0.25 (0.03)
South Africa	0.03	0.98	0.96	0.17 (0.05)
Pooled weighted	0.021	1.00	0.98	0.40 (0.01)

[1] Model fit criteria were all good (RMSEA ≤ 0.05, CFI ≥ 0.95 and TLI ≥ 0.95), except for RMSEA and TLI for the path model of Brazil, which were fair (RMSEA = 0.07, CFI ≥ 0.95, TLI = 0.93).

ONLINE SUPPORTING MATERIAL

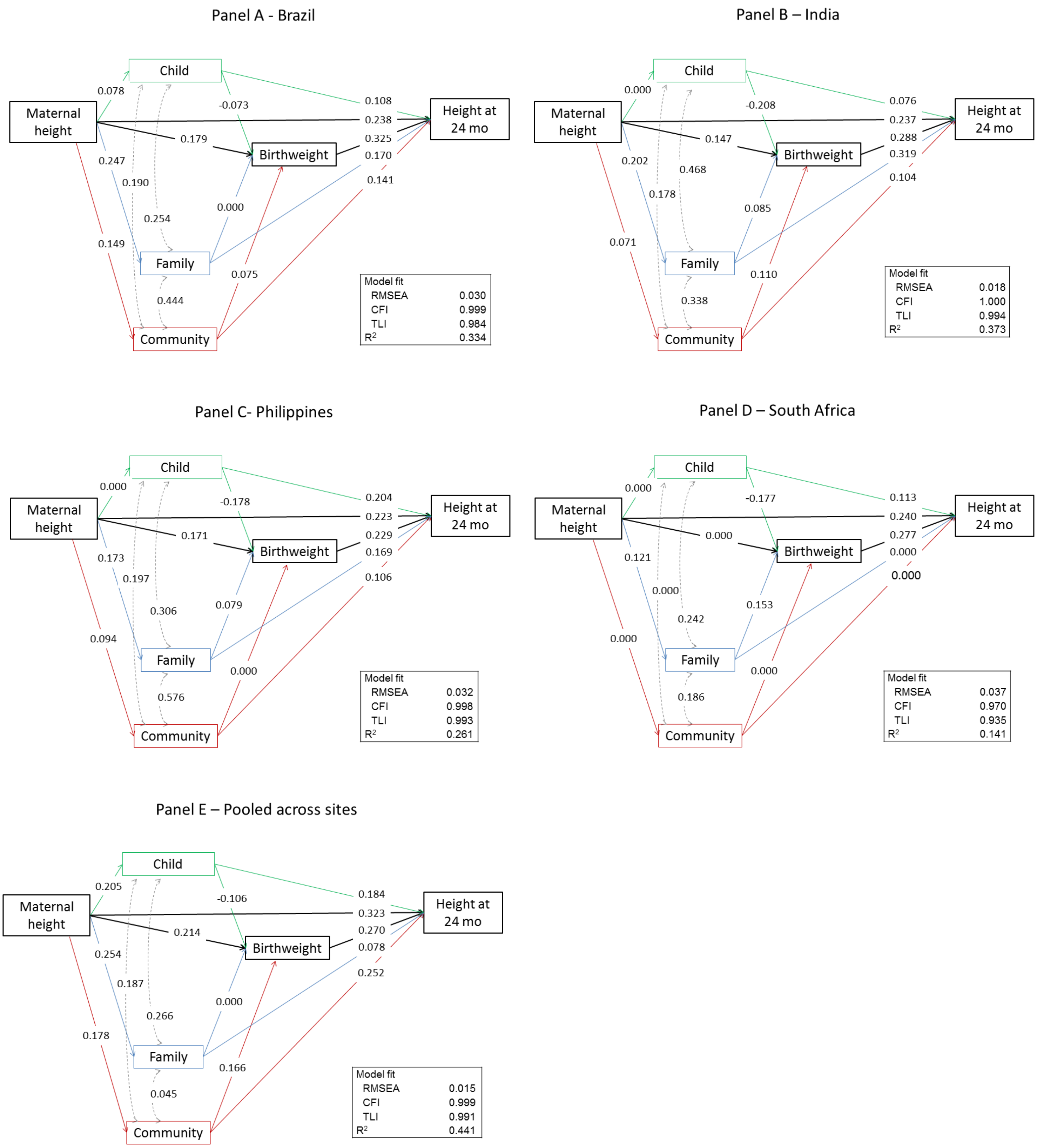

SUPPLEMENTAL FIGURE 1. Panels A to E: Pathways to child height at 24 months in Brazil, India, Philippines, South Africa and pooled across sites.
